# Infrared Thermography Imaging for Assessment of Peripheral Perfusion in Patients with Septic Shock

**DOI:** 10.3390/bioengineering10060729

**Published:** 2023-06-18

**Authors:** Sigita Kazune, Edgars Vasiljevs, Anastasija Caica-Rinca, Zbignevs Marcinkevics, Andris Grabovskis

**Affiliations:** 1 Department of Anesthesiology, Riga Stradins University, LV-1007 Riga, Latvia; 2 Laboratory of Biophotonics, Institute of Atomic Physics and Spectroscopy, University of Latvia, LV-1004 Riga, Latvia; 3 Residency Development Department, University of Latvia, LV-1004 Riga, Latvia; 4 Department of Human and Animal Physiology, Faculty of Biology, University of Latvia, LV-1004 Riga, Latvia; andris.grabovskis@lu.lv

**Keywords:** infrared thermography, skin temperature, lower limb, peripheral perfusion, circulatory shock, sepsis

## Abstract

Skin temperature changes can be used to assess peripheral perfusion in circulatory shock patients. However, research has been limited to point measurements from acral parts of the body. Infrared thermography allows non-invasive evaluation of temperature distribution over a larger surface. Our study aimed to map thermographic patterns in the knee and upper thigh of 81 septic shock patients within 24 h of admission and determine the relationship between skin temperature patterns, mottling, and 28-day mortality. We extracted skin temperature measurements from zones corresponding to mottling scores and used a linear mixed model to analyze the distribution of skin temperature in patients with different mottling scores. Our results showed that the distribution of skin temperature in the anterior thigh and knee is physiologically heterogeneous and has no significant association with mottling or survival at 28 days. However, overall skin temperature of the anterior thigh and knee is significantly lower in non-survivors when modified by mottling score. No differences were found in skin temperature between the survivor and non-survivor groups. Our study shows the potential usefulness of infrared thermography in evaluating skin temperature patterns in resuscitated septic shock patients. Overall skin temperature of the anterior thigh and knee may be an important indicator of survival status when modified by mottling score.

## 1. Introduction

Sepsis is a life-threatening syndrome with high mortality rates [1]. Despite acceptable hemodynamic values achieved during resuscitation in patients with septic shock, microcirculatory perfusion of organs can still be variable [2,3]. To improve outcomes and allow better prognostication, hemodynamic monitoring should be directed towards the early detection of microcirculatory hypoperfusion. It has been suggested that reduction of peripheral perfusion could be an early marker of microcirculatory hypoperfusion in septic shock [4,5,6].

Changes in skin temperature and color have long been used as simple and reliable tools to assess peripheral perfusion. Subjective assessment of skin temperature and the difference in skin temperature between a distal and proximal skin zone have been investigated in the evaluation of patients with septic shock. Cool skin and large skin temperature gradients have been associated with more severe organ failure and poor survival in patients with sepsis [7,8].

Infrared thermography can be used to visualize and quantify the infrared radiation emitted from any surface. It allows the acquisition of highly accurate, color-coded images showing the temperature variation over the surface of the skin. As well as allowing accurate measurements of temperature gradients across an extremity, the full pattern of temperature distribution (and therefore peripheral perfusion) can be evaluated. Although it could be a useful tool for the assessment of peripheral perfusion, there is little research regarding its use in septic shock patients. Ferraris et al. investigated the link between the presence and absence of mottling and skin temperature using infrared imaging [9]. Patients with mottling were found to have a lower skin temperature than patients without mottling. However, the patterns of temperature distribution in septic shock patients in relation to mottling scores and mortality have not been investigated. Potentially, infrared thermography could be used to detect the thermal pattern of mottling similar to heterogenous discoloration of the skin and possibly present in the same areas.

Our study aimed to map thermographic patterns in the knee and upper thigh in resuscitated patients with septic shock using infrared thermography. We hypothesized that by using thermographic imaging, we would observe different patterns of skin temperature distribution in patients with and without mottling. We hypothesized that these patterns would be associated with mottling scores and poor outcomes.

## 2. Materials and Methods

This prospective observational study was conducted in a 16-bed mixed intensive care unit (ICU) of a tertiary university hospital from March 2017 to February 2019. The study protocol was approved by the Institutional Ethics Committee (26/23.02.2017) and each participant or their relative gave informed consent. Consecutive patients admitted to the unit were screened for septic shock, defined according to international consensus definitions for sepsis and septic shock [10]. Patients were eligible for inclusion if they were older than 18 years and required a vasopressor infusion to maintain a mean arterial pressure > 65 mmHg despite adequate volume resuscitation. Exclusion criteria were severe agitation, advanced malignancy and an inflammatory process, and bruising or scarring in the knee or upper leg area.

Management of the sepsis patients was not affected by thermographic imaging and was decided by a physician not involved in the study. It was guided by local protocols that included fluid administration and vasopressor infusion to maintain hemodynamics (mean arterial pressure > 65 mmHg) and included standard laboratory tests, invasive arterial monitoring, a central venous catheter, and ventilatory support if needed.

All patients were recruited into the study within 24 h of admission to the ICU after initial resuscitation was achieved and there had been no change in vasopressor requirements for at least 1 h. The study collected data on core temperature, heart rate, arterial pressure, and central venous pressure in all patients. These vital signs were monitored using the Philips IntelliVue MP70 patient monitor with Multimeasurement Module (Philips, Eindhoven, The Netherlands), which is the standard of care in the hospital’s ICU. The sampling rate for the numeric values was 1 Hz. Heart rates were calculated from the electrocardiogram, while core temperature was measured using a commercially available temperature measurement probe (Reusable esophageal/rectal temperature probe, Philips, Eindhoven, The Netherlands). This probe was placed in the esophagus and interfaced with the monitor through the M1029A temperature module. The accuracy of the probe was ±0.2 °C at 40 °C, according to the manufacturer’s specifications. For monitoring oxygen saturation, a pulse oximeter with a reusable glove sensor (model M1191B, Philips Medical Systems, BG Eindhoven, The Netherlands) was attached to the patient’s finger, from which a photoplethysmogram signal was recorded and oxygen saturation computed. For the measurement of invasive pressures, arterial and central venous catheters were connected to a disposable pressure transducer (Combitrans, Braun, Kronberg im Taunus, Germany). These transducers were calibrated to the level of the patient’s heart, following the standard procedure recommended by the manufacturer.

Demographic characteristics (sex, age), admission diagnosis, arterial lactate levels, and vasopressor administration rate were recorded. Mottling of the thigh and knee was visually assessed on both legs. The Mottling score, which describes the extent of the mottled area, was determined on a 6-point scale ranging from 0 to 5, as previously described [11]. The severity of the disease was measured by Acute Physiology and Chronic Health Evaluation (APACHE) II [12] and Sequential Organ Failure Assessment (SOFA) [13] scores. Survival status was recorded at 28 days. Primary outcomes were skin temperatures of predefined zones on the knee and thigh of each patient obtained using thermographic imaging.

The room temperature in the ICU was controlled at 22 ± 0.5 °C and was checked before each thermographic imaging session. The blanket covering the patient’s legs was removed 10 min before measurement for acclimatization to the ambient temperature. Thermographic images of the anterior surface of the upper leg and the knee of one leg were taken for all participants. Thermal images were recorded using a FLIR A600 (FLIR systems, Täby, Sweden) thermal imaging camera. The camera was placed on a tripod at a distance of 1 m above the right upper leg, perpendicular to the plane of acquisition. The upper leg from the groin to the inferior edge of the patella was captured in a thermographic image with the patient in the supine position with straight legs. A second thermographic image was taken in a supine position with the legs straight from the dorsal aspect of the feet to assess the temperature of the big toe.

**Figure 1:**
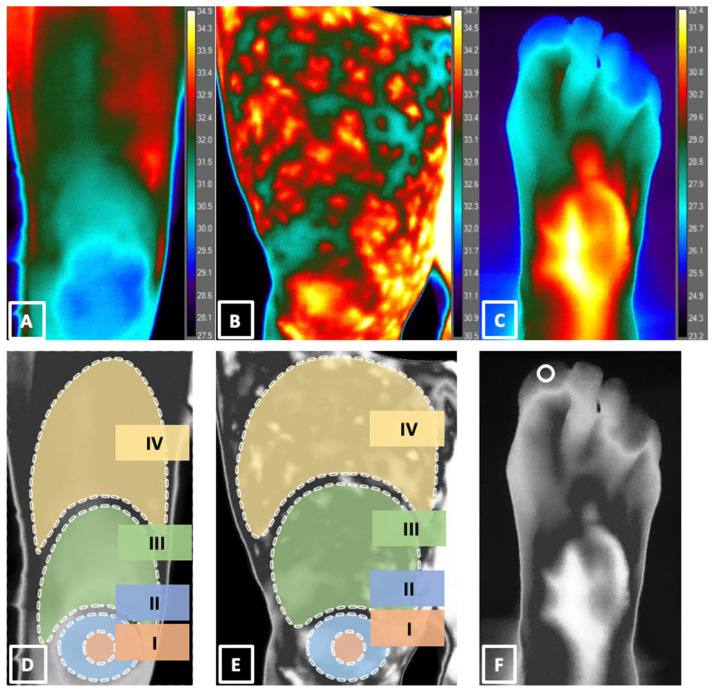
(**A**,**B**) Thermographic images of anterior thigh and knee; (**C**) thermographic image of the dorsal aspect of the foot; (**D**,**E**) temperature measurement zones defined to match mottling scores 1 to 4, Zone I, coin-sized area located in the center of patella, Zone II, area over the entire patella, Zone III, area up to the middle of upper leg, and Zone IV, area up to the inguinal fold; (**F**) temperature measurement zone on the toe.

Measurement zones were predefined to match the mottling score regions described by Ait-Oufella et al. [11] and given roman numeral identifiers I to IV (Figure 1). Thermographic temperature measurements in each zone of each patient were extracted manually with FLIR ResearchIR MAX 4 (4.40.11.35) software by using an ellipse tool on the region of the knee area, which corresponds to a mottling score of 1 (4000 pixels) and 2 (20,000 pixels). The upper leg zones corresponding to mottling scores of 3 and 4 were analyzed by the polygonal lasso tool, with pixel areas varying in size depending on the size of the upper leg. To measure the temperature of the big toe, the area was selected using the ellipse tool from the skin of the toe above the nail (300 pixels). The mean temperature values for each area were calculated by the summation of temperature in every pixel of the defined area, and then the result was divided by the number of summed pixels, and temperatures for each zone for each patient were tabulated.

All statistical analyses were performed in version R 4.0.5 (R Core Team 2021). Continuous variables were expressed in median and interquartile ranges (IQR). We evaluated the differences between groups of patients using the Kruskal–Wallis test. Categorical variables were presented as counts and proportions. A *p* value < 0.05 was considered statistically significant.

To analyze the distribution of skin temperature over the knee and thigh in patients with different mottling scores, we applied a mixed linear model that included the measurement zone, mottling score, and the interaction of the measurement zone and mottling score as fixed effects. To test the significance of fixed effects and interactions, we used likelihood ratio tests to compare a model including the effect of interest with a nested model with this effect of interest removed. The interaction between measurement zone and mottling score was not significant, so we removed the interaction term from the model. We performed an additional model with measurement zone, mottling score, 28-day survival status, survival status, and mottling score interaction as fixed effects, and patient identity as a random effect. To account for the correlation as well as possible differences in variances/standard deviations in repeated temperature measurements in the same patient, we assumed an unstructured covariance pattern.

## 3. Results

A total of 142 patients were assessed for eligibility, and 81 were included in the study. Of the 81 included patients, 56 had no mottling, and eight, fourteen, and three had a mottling score of 1, 2, and 3, respectively. There were no patients with a mottling score of 4 or 5. We were able to perform thermography of the dorsal aspect of the foot only in 57 cases due to dressings and heel pressure ulcer prophylaxis measures [14].

Baseline patient characteristics are given in Table 1. A significant difference in observed 28-day mortality was found between patients with a mottling score of 2 or more compared to patients with no mottling (*p* = 0.001). The groups with different mottling scores did not differ in other demographic or perfusion variables. No correlation was found between knee and thigh skin temperatures and the temperature of the big toe.

**Figure 2:**
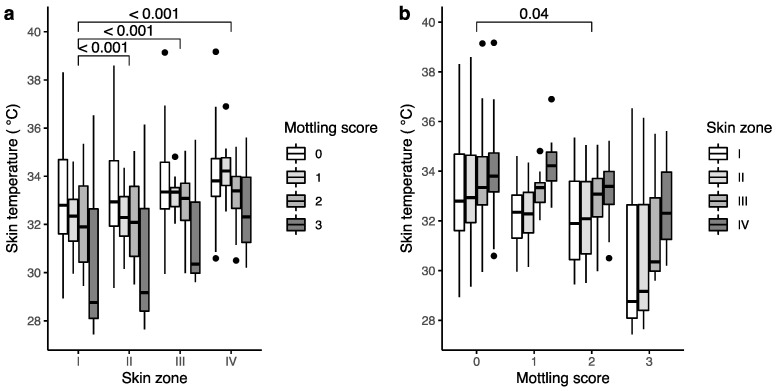
Skin temperature variation among septic shock patients by mottling score and measurement zone. (**a**) Skin temperature by measurement zone with significant model effects; (**b**) skin temperature by mottling score with significant model effects. Observations lying beyond 1.5 times the interquartile range are shown as black dots. Full model results are shown in Supplemental Table S1.

The distribution of skin temperature in the anterior knee and thigh by mottling score in measurement zones I to IV is shown in Figure 2a. Depending on the measurement zones compared, 74.5 to 99.5% of the variation in skin temperature came from variation between subjects. Nonetheless, a significant skin temperature difference was found between the different measurement zones (*p* < 0.0001; Table 2; Supplemental Table S1). The effect of mottling on skin temperature in the entire patient group did not reach statistical significance. However, a mottling score of 2 was associated with a significant decrease in skin temperature of 0.9 °C (95% CI: −1.7 to 0, *p* = 0.04) compared to patients without mottling, as shown in Figure 2b and Table 2. Although skin temperatures in patients with a mottling score of 3 were lower compared to patients with no mottling, statistical significance was not reached, possibly because of the small number of patients in this group. The interaction between mottling score and skin temperature distribution was not significant. No association was found between mottling score and big toe temperature.

**Figure 3:**
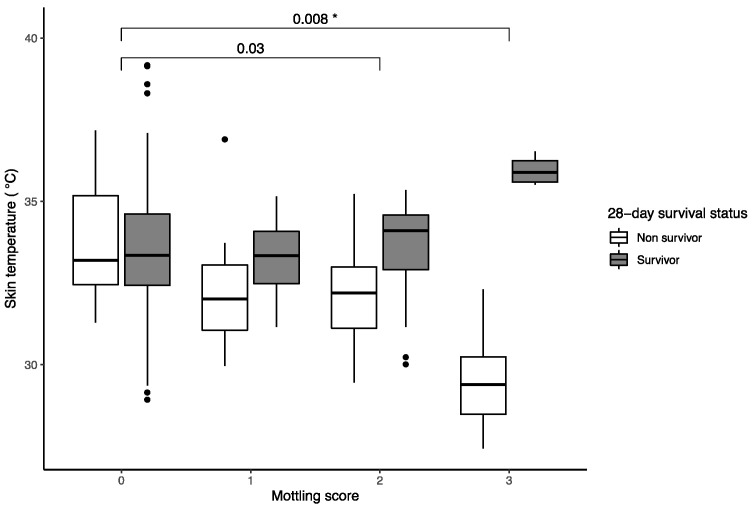
Variation in skin temperature among patients with septic shock by mottling score and survival status at 28 days with significant model effects. Asterisks denote significant interaction: * *p* < 0.05. Observations lying beyond 1.5 times the interquartile range are shown as black dots. Full model results are shown in Supplemental Table S2.

Of the 81 patients included, 22 died within 28 days of admission. There was no association between skin temperature in the knee and upper leg and 28-day mortality (*p* = 0.18; Table 3), but skin temperature was significantly affected by survival status and mottling score interaction (*p* = 0.01; Figure 3, Supplemental Table S2).

Survivors with mottling scores of 2 and 3 had significantly higher skin temperatures than non-survivors (Table 3). The median big toe temperature of patients who died was 26.0 (24.7–27.0) °C, whereas the median big toe temperature of patients who survived was 31.0 (27.2–34.0) °C. The association between survival and big toe temperature was significant (odds ratio 1.3 per °C; 95% CI 1.28–1.33; *p* = 0.002).

## 4. Discussion

This study reports the pattern of temperature distribution in the skin of the knee and upper thigh obtained by infrared thermography in resuscitated patients with septic shock and relates these patterns to mottling score and 28-day survival. We were unable to show differences in skin temperature distribution between patients with differing mottling scores, but non-survivors had lower skin temperatures if mottling was present.

Multiple studies have explored skin temperature or temperature gradients for non-invasive monitoring of peripheral perfusion in critically ill patients, mostly using contact thermometers [15,16]. To our knowledge, there is only one other study that has assessed thigh and knee skin temperatures in septic shock in relation to mottling score [9]. In their analysis, Ferraris et al. compared pooled skin temperatures by mottling zone between groups with different mottling scores. As shown by the significant baseline temperature variation between individuals found in our study, the pattern of temperature distribution is superimposed onto the baseline value of each particular patient, and repeated temperature measurements in each individual are correlated. The choice of linear mixed-effects modeling allowed us to account for baseline variability and explore patterns of temperature variation rather than values in separate skin zones across mottling scores.

The use of infrared thermography has been explored in a number of conditions characterized by changes in peripheral perfusion [17], but there is surprisingly little data available regarding knee and thigh skin temperature ranges and variations in healthy individuals [18,19]. Zaproudina et al. recorded whole-body skin temperature under ambient conditions of 23–24 °C in sixteen healthy young men, with median skin temperatures for the big toe, knee, and anterior thigh area of 22.3, 29.1, and 30.3 °C, respectively. In our patient group, the corresponding temperatures were 26.4, 32.7, and 33.3 °C. Zaproudina et al.’s thermographic data show similar or even lower skin temperatures in healthy individuals compared to our and Ferraris et al.’s septic shock cohorts. This observation raises questions about the usefulness of absolute skin temperature values and temperature assessment by touch in patients with septic shock to differentiate between normal and abnormal peripheral perfusion. Nevertheless, within the range of temperature ranges similar to those of healthy volunteers, an association between a higher mottling score and a lower skin temperature in patients with septic shock was found in our study and in the study by Ferraris et al. [9].

Increased skin temperature gradients and an increased difference in temperature between a proximal and distal temperature measurement site are thought to be more accurate reflections of abnormal peripheral blood flow than absolute skin temperature values [8,9]. A decreasing skin temperature from the groin to the knee area has been described in healthy volunteers [16] and is thought to reflect thermoregulatory vasoconstriction [20]. In our study, there was a very similar pattern of skin temperature distribution with lower temperatures in the knee area (mottling zones I and II) and higher temperatures in the upper thigh area (mottling zones III and IV), and the temperature distribution pattern remained the same in patients with mottling scores 0–3. Previous studies [7,8] have included assessments of knee-to-room and core-to-knee temperature gradients in septic shock patients but have reported only toe and index finger temperature gradients, as these were found to be stronger associated with mortality. Due to the abundance of arteriovenous anastomoses in the skin of the hands and feet [21], the effects of reduced blood flow on skin temperature might not be applied in the same way to the thigh and knee skin areas [22].

Even in resuscitated patients with circulatory shock, subjectively and objectively cool skin has been linked to poor outcomes [5,7,23], though in studies with septic shock cohorts, results have been inconclusive [24,25]. In our study, non-survivors had lower skin temperatures only if mottling was present. A previous study by Hernandez et al. has suggested that several indicators of peripheral perfusion, such as temperature gradients and capillary refill time, should be interpreted together [24]. Our findings, using a different combination of mottling and skin temperature, further support the value of joint interpretation.

In our opinion, the skin temperature of the thigh and knee areas cannot directly represent skin perfusion. First, even if hypoperfusion affects skin temperature, continuous equilibration of the thermal gradient would possibly conceal the thermographic representation of mottling [11]. Secondly, skin perfusion depends on the abundance of arteriovenous anastomoses. The relative lack of these anastomoses in the skin of the thigh and knee does not allow the application of thermographic findings to the hands and feet directly [14]. The results of our and previous studies [9] show that the mottling score is associated with a lower skin knee area temperature, which could be due to perfusion abnormalities in the region supplied by the genicular arteries [26]. Decreased perfusion in this area could be explained by increased systemic vascular resistance and decreased cardiac output. Indeed, an increased thigh–knee temperature gradient has been shown to be associated with low cardiac output in cardiac patients [27,28].

In this study, we utilized classical infrared thermography to evaluate thermal patterns, as it can be easily integrated into the existing workflow in intensive care units. However, the implementation of active dynamic thermography (ADT), an advanced version of infrared thermography, holds the potential for significant improvements. ADT involves the application of a thermal challenge to the area of interest and recording the rate and pattern of temperature changes until equilibrium is reached. This technique offers enhanced image contrast and has the potential to serve as a superior indicator of perfusion in the skin and subcutaneous tissue [29,30]. Nonetheless, to fully capitalize on the benefits of ADT, it is crucial to address safety concerns associated with applying cold stimuli to patients with pre-existing peripheral perfusion issues, such as patients with circulatory shock.

Our study has several limitations. To reduce the variability of skin perfusion during ongoing resuscitation, we obtained thermographic images after initial hemodynamic stabilization. As a result, no patients with a mottling score of 4 or 5 were available for inclusion in our study, resulting in uncertain skin temperature data in this high-risk population. Unfortunately, cardiac output data were not available for most patients and were not collected, so there are no data to link cardiac output with skin temperature in the thigh and knee.

The unanswered question remains as to whether mottling and changes in skin temperature in the knee area and anterior thigh arise from the same microcirculatory perfusion disorder or are independent markers of peripheral perfusion. This study points towards the latter hypothesis, but a larger cohort of patients should be studied.

## 5. Conclusions

The presence and extent of mottling were not associated with changes in the distribution of skin temperature in the knee and thigh in resuscitated patients with septic shock. A lower overall skin temperature was associated with a high mottling score (≥2), especially in non-survivors.

## Figures and Tables

**Table 1:** Demographic, clinical, and hemodynamic characteristics of study subjects.

Parameter	Value
Age (years)	72 (62, 79)
Female (*n* (%))	33 (40.7%)
APACHE II	23 (18, 27)
SOFA	9 (7, 12)
Mean arterial pressure (mm Hg)	75.0 (67, 85)
Norepinephrine dose (mcg/kg/min)	0.11 (0.08, 0.17)
Serum lactate (mmol/L)	2.6 (1.9, 4.9)
Temperature of the big toe (°C)	26.4 (23.6, 32.3)
Core to knee temperature difference (°C)	3.3 (2.2, 4.4)
Core to toe temperature difference (°C)	6.2 (2.8, 9.5)
28-day mortality	22 (27.2%)

Data are presented as numbers with percentages (%) or medians with interquartile range APACHE II, Acute Physiology, Age, Chronic Health Evaluation II; Sequential Organ Failure Assessment (SOFA) score represents maximum value calculated within 24 h of intensive care unit admission.

**Table 2:** General linear mixed model estimates for fixed effects of skin zone and mottling score on skin surface temperature per patient.

	Estimate	Lower 95% CI	Upper 95% CI	*p* Value
(Intercept)	32.99	32.47	33.52	>0.001
Skin zone				
I (ref)				
II	0.12	0.07	0.17	>0.001
III	0.64	0.42	0.87	>0.001
IV	1.04	0.72	1.37	>0.001
Mottling score				
0 (ref)				
1	0.19	−0.87	1.24	0.73
2	−0.86	−1.69	−0.02	0.04
3	−1.39	−3.05	0.26	0.10

CI: confidence interval; ref: reference category.

**Table 3:** General linear mixed model estimates for fixed effects of survival status and mottling score on skin surface temperature per patient.

	Estimate	Lower 95% CI	Upper 95% CI	*p* Value
(Intercept)	34.77	32.58	34.53	>0.001
Survivor	−0.67	−1.65	0.31	0.18
Mottling score				
0 (ref)				
1	1.05	–1.06	3.16	0.33
2	−1.89	−3.17	−0.62	>0.001
3	−3.03	−5.14	−0.92	>0.001
Survival status and mottling score interaction				
Survivor: MS0 (ref)				
Survivor: MS1	−1.11	−3.52	1.30	0.37
Survivor: MS2	1.84	0.04	3.64	0.04
Survivor: MS3	4.09	0.65	7.54	0.02

CI: confidence interval; ref: reference category; MS: mottling score.

## Data Availability

All the data supporting our findings are available from the corresponding author upon reasonable request.

## Supplementary Materials

The following supporting information can be downloaded at https://www.mdpi.com/article/10.3390/bioengineering10060729/s1: Table S1: General linear mixed model tests the effects of measurement zone and mottling score on skin surface temperature; Table S2: General linear mixed model tests the effects of measurement zone, 28-day survival status, and mottling score on skin surface temperature.
