# Supplementary material for: Infrared Thermography Imaging for Assessment of Peripheral Perfusion in Patients with Septic Shock
Source: Bioengineering (Basel). 2023 Jun 18;10(6):729. doi: 10.3390/bioengineering10060729 (PMC10295664; doi:10.3390/bioengineering10060729)
Supplement: Supplementary file 1 [file bioengineering-10-00729-s001.zip › bioengineering-2444112-supplementary.pdf]

bioengineering

MDPI

Supplementary Materials

Infrared Thermography Imaging for Assessment of Peripheral Perfusion in Patients with Septic Shock

Sigita Kazune [1,2,*], Edgars Vasiljevs [3], Anastasija Caica-Rinca [2,4], Zbignevs Marcinkevics [2,4] and Andris Grabovskis [4]

1 Department of Anesthesiology, Riga Stradins University, LV-1007 Riga, Latvia
2 Laboratory of Biophotonics, Institute of Atomic Physics and Spectroscopy, University of Latvia, LV-1004 Riga, Latvia
3 Residency Development Department, University of Latvia, LV-1004 Riga, Latvia
4 Department of Human and Animal Physiology, Faculty of Biology, University of Latvia, LV-1004 Riga, Latvia; andris.grabovskis@lu.lv
* Correspondence: sk13171@edu.lu.lv

Table S1. General linear mixed model tests the effects of measurement zone and mottling score on skin surface temperature.

Variable	F	df	P
Intercept	46457.31	1317	**<0.0001**
Measurement zone	14.48	3317	**<0.0001**
Mottling score	2.27	3317	0.08

Degrees of freedom (df; numerator, denominator) are shown for the F statistic. Bold P values indicate significant effects (α = 0.05)

Table S2. General linear mixed model tests the effects of measurement zone, 28-day survival status and mottling score on skin surface temperature.

Variable	F	df	P
Intercept	49943.78	1313	**<0.0001**
Measurement zone	14.48	3313	**<0.0001**
Survival status	0.73	1313	0.40
Mottling score	2.46	3313	0.06
Survival status × mottling score	3.57	3313	**0.01**

Degrees of freedom (df; numerator, denominator) are shown for the F statistic. Bold P values indicate significant effects (α = 0.05)
